# Cancer Metabolism and Drug Resistance

**DOI:** 10.3390/metabo5040571

**Published:** 2015-09-30

**Authors:** Mahbuba Rahman, Mohammad Rubayet Hasan

**Affiliations:** 1 Sidra Medical & Research Center, P.O. Box 26999 Doha, Qatar; 2 Weill Cornell Medical College in Qatar, P.O. Box 24144 Doha, Qatar; E-Mail: mhasan@sidra.org

**Keywords:** drug resistance, metabolic pathways, systems biology, metabolic flux analysis, antimetabolites, targeted therapy

## Abstract

Metabolic alterations, driven by genetic and epigenetic factors, have long been known to be associated with the etiology of cancer. Furthermore, accumulating evidence suggest that cancer metabolism is intimately linked to drug resistance, which is currently one of the most important challenges in cancer treatment. Altered metabolic pathways help cancer cells to proliferate at a rate higher than normal, adapt to nutrient limited conditions, and develop drug resistance phenotypes. Application of systems biology, boosted by recent advancement of novel high-throughput technologies to obtain cancer-associated, transcriptomic, proteomic and metabolomic data, is expected to make a significant contribution to our understanding of metabolic properties related to malignancy. Indeed, despite being at a very early stage, quantitative data obtained from the omics platforms and through applications of ^13^C metabolic flux analysis (MFA) in *in vitro* studies, researchers have already began to gain insight into the complex metabolic mechanisms of cancer, paving the way for selection of molecular targets for therapeutic interventions. In this review, we discuss some of the major findings associated with the metabolic pathways in cancer cells and also discuss new evidences and achievements on specific metabolic enzyme targets and target-directed small molecules that can potentially be used as anti-cancer drugs.

## 1. Introduction

Cancer remains one of the most challenging medical problems in the world, despite the fact that investments and efforts to find ways to cure cancer in recent decades were massive [1,2]. Globally, an estimated, 14.1 million new cancer cases and 8.2 million cancer-associated deaths were reported in 2012 [3]. Although significant progress has been made in understanding the genetic and epigenetic factors that promotes tumor growth and metastasis, the concomitant progress in developing new cancer drugs is much slower. Currently available treatment options for cancer include surgery, radiotherapy, endocrine therapy, immunotherapy, and chemotherapy. While conventional chemotherapy is still considered as the first line of treatment for many types of cancers, treatment failures are common, because of drug resistance [4,5]. In fact, chemotherapy resistance is blamed for being one of the most important contributors of high mortality in cancer. Furthermore, most chemotherapy drugs are cytotoxic and non-specific, and therefore, associated with a wide-range of side-effects [6,7]. To circumvent the problems associated with the use of chemotherapeutic drugs, treatment protocols have been modified so that a combination of drugs with different molecular targets is used. The benefit of this approach is that it reduces the risk of clonal selection of resistant tumor cells to one drug only. Moreover, each drug can be applied at a lower dose to minimize side-effects [8]. These drug combinations include both conventional, cytotoxic drugs as well as novel, targeted drugs that interferes with abnormal cellular signaling in cancer. Recently, targeted therapies for cancer have received much attention because of the expectation that they will be more effective and less harmful than systemic chemotherapy. The goals of targeted therapy is to specificity block or modify the functions of molecular targets such as growth factors, cell surface receptors or enzymes that are associated with tumor-genesis and cancer progression [8,9]. Apart from these intrinsic and extrinsic factors, altered metabolic factors and pathways of cancer cells have also been suggested as important targets for the development of novel, combinatory drugs to improve response to conventional chemotherapy [10,11,12,13,14].

The metabolic pathways of a cell consist of a network of interacting genes, proteins and metabolite reactions [15]. Their functions are carried out by organized and hierarchical levels of information, which are controlled by intricate regulatory structures, mostly proteins and signaling molecules. This complex network provides dynamicity and robustness to the cell, offering the opportunity to dissect the complexity of the cell as a system and relate the genotype to its phenotype. These properties are regulated at several checkpoints in normal cells. However, in cancer cells, many of these regulatory networks are dysregulated and associated with uncontrolled growth and proliferation [15,16]. In fact, cancer cells adopt alternative metabolic pathways, and this has been reported by Otto Warburg almost 90 years ago [17]. At present, altered metabolism is considered as one of the hallmarks of cancer cells, and few studies showed that metabolic alterations are also linked to cancer drug resistance [12]. The altered metabolism is not caused by a single event. Instead, these are caused by multiple events, where the systems of the cells, comprised of genes, proteins and metabolites act in concert, to generate a certain cancer phenotype. Therefore, deciphering the underlying causes of disease pathogenesis and response to therapeutic targets require a holistic approach, like systems biology, in order to identify new molecular targets to treat disease or to correct altered metabolic networks in cancer [13,15]. In this review, we will briefly discuss the technologies, especially systems biology approaches that are used to identify dysregulated metabolic pathways in cancer cells followed by findings on the drug resistance phenotypes that are associated with altered metabolic pathways in cancer cells, and finally discuss some of the related, anticancer drugs that are currently at the clinical or pre-clinical stages of development.

## 2. Technologies Used to Study the Metabolic Pathways of Cancer Cells

Revolutionary development in massively parallel, DNA sequencing technology has given an enormous opportunity to the cancer researchers to identify and understand the genetic and epigenetic factors that work in the tumor microenvironment [18]. It is now evident that differential expression of a gene in the tumor microenvironment not only affects a particular type of signal, but affects multiple pathways at various levels such as genes, proteins and metabolites. Analytical techniques have been developed that allowed simultaneous quantification of a large variety of molecules such as mRNAs and proteins. In general terms, these techniques are called “high-throughput (HT) omics technologies,” which belongs to the field of systems biology [15].

**Figure 1:**
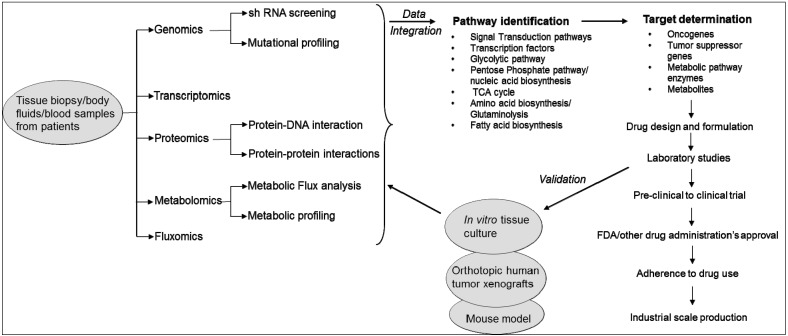
Systems biology approach to study cancer metabolism and identification of therapeutic targets.

In fact, the birth and growth of the field of systems biology has been driven by technological innovations in high-throughput techniques and omics technologies used in life sciences research. These include genomics, transcriptomics, proteomics, metabolomics and fluxomics [19]. Systems biology consists of both “wet” experiments and “dry” experiments. In “wet” experiments, techniques are applied for the acquisition of quantitative or semi-quantitative data on the expression levels of genes, proteins and metabolites from the clinical specimens (e.g., blood, serum, urine, *etc*.) or other samples being analyzed. While wet experiments enable data acquisition, the “dry” experiments perform data analysis using computational and mathematical tools and models. It has three phases: (i) integrate high throughput data; (ii) generate hypothesis on the biological pathways; and (iii) design experiments to evaluate the *in silico* predictions. The ultimate goal of the “dry” experiments is to construct hypothetical models that closely reflect the “phenotype” of the samples used in wet experiments. Results obtained from the analysis are further validated in different immortalized cell lines or tissue cultures or mouse models (Figure 1) [19].

Systems biology approaches are currently used in cancer research to understand the effects of drugs on cell signaling networks [20]. The approach is also used for the identification of biomarkers for diagnostics and selection of drug targets, identify specific molecular mechanisms for different types of diseases or cancers, and determine progression stage of a disease and classification of diseases or cancers. Different omics platforms provide data at different levels of the cell or the system. These high-throughput data are comprehensive and more un-biased than the reductionist approaches, where one-on-one biological data are used [20]. However, almost all of the omics technologies used in systems biology generate large data set. Analyzing the large data set is now the bottleneck of systems biology because real signals or molecular mechanisms and biological principles are buried under these large datasets. In addition, inconsistency arises as different groups use different computational models to interpret the data. Furthermore, these analyses cannot always distinguish small fold changes. Cancer cells show a great level of heterogeneity in their genomes. Therefore, to elucidate the molecular basis of cancer and the drug resistance phenotype, it requires a systematic investigation of metabolic fluxes, enzymes and oncogenes to identify critical reactions in cancer cells [19,20]. An expression change in a metabolic enzyme or metabolite of only 1 or 2 fold is often sufficient to substantially alter the rate of a metabolic reaction. High throughput methods need to be complemented with methods that can detect the metabolic state of a cell. In this context, “metabolomics”, which is also a part of the systems biology, measures and interprets the time-related concentration and flux of endogenous metabolites in cells [21,22].

An integral part of metabolomics is metabolic flux analysis. Metabolic flux is defined as the rate of inter-conversion between two chemical species taking part in a reaction known as metabolism. In other words, metabolism is an integrated term of all reactions that provide energy to living organism, and metabolic pathways are series of reactions linked by intermediaries within metabolism. Metabolic fluxes reflect the final outcome of the cellular orchestration under defined genetic and environmental or diseased or treated conditions [15]. Metabolomics links the physiological knowledge from genotype to phenotype, by obtaining an instantaneous snapshot of final gene products, which in this case, are metabolites. In metabolomics studies, extracellular metabolite profiles capture the features of nutrient uptake and metabolite secretion. On the other hand, intracellular metabolites reflect gene functions in complex cell metabolism. The intracellular metabolite profiles elucidate specific cellular processes and thereby the data can be used for comparative analysis of metabolite profiling from mutant species. Metabolomics can also reveal enzyme activities, silent genes and metabolic network topology [21,23].

Metabolite detection relies on the availability of analytical instruments such as NMR spectrometers, mass spectrometers or HPLC. Nowadays, metabolomics has reached a high level in terms of sensitivity, reproducibility, metabolite-coverage and sample-throughput. Metabolomics is also an excellent choice for experimental verification because most of the analytical methods of systems biology generate hypothetical models [24]. Metabolite homeostasis in health or the dynamic metabolic responses of cells, tissues and organisms to various environmental factors, toxic factors, genetic modifications or diseases is now investigated through the application of metabolomics. Metabolomics studies are performed with multiple aims including: (i) detection, identification, annotation, and quantification of metabolites; (ii) differentiation of native metabolites from artifacts; (iii) identification of biomarkers; (iv) categorization of metabolites or signature molecules; (v) resolution of spatial or temporal metabolomes; (vi) determination of metabolic phenotypes for genome-wide association studies (GWAS); (vii) metabolic pathway analysis and prediction; (viii) identification of linkages between pathways; and (ix) analysis of disease or drug action mechanisms to facilitate diagnostics [24]. However, these aims cannot be realized simultaneously due to technological restrictions. Furthermore, the size of different metabolomes differs among different species and their identification is a tedious process [24].

Although the concentration of extracellular metabolites or primary metabolites such as amino acids, organic acids and sugar phosphates can be detected with the existing instruments, intracellular metabolites are generated at such a low concentration that they may not be detectable easily [21]. Some of the notable limitations of the application of metabolomics are: (i) the loss and degradation of metabolites during sample quenching and separation; (ii) difficulties in the detection of putative isobaric or isomeric metabolites; (iii) inability of direct metabolite profiling to reveal the actual enzyme activities; and (iv) the high cost of high resolution MS [21]. The approach also requires extreme accuracy as these has to be parallelized between many laboratories worldwide. Equipment like ultra-high performance liquid chromatography coupled with fourier transform ion cyclotron resonance mass spectrometry (UHPLC-FT-ICR-MS), nuclear magnetic resonance (NMR) or liquid chromatography mass spectrometry (LC-MS) are used to detect metabolites, which are universally applicable from technical point of view [20,24]. Since a metabolite pool is regulated by its carbon fluxes, *i.e.*, enzyme reaction rates, isotope tracers are introduced to track the fate of metabolites and functional pathways to overcome the problems discussed above [11,21].

Isotopes like radiotracers have been applied to study enzyme reactions for a long time. The disadvantage of using isotopes is that all reactions do not incorporate or release isotopes, disabling the calculation of true fluxes due to unknown specific activities of precursor pools. Due to the limitation of the radioactive isotopes, stable isotopes were introduced, which allowed the calculation of true fluxes from the labelling patterns of metabolites of interest [21]. Different types of tracers are available, such as ^2^H, ^13^C or ^15^N. The choice of the tracer will affect the information obtained for particular biosynthetic fluxes, for example, ^2^H is viewed more as a universal tracer to determine *in vivo* and *in vitro* turnover rates of macromolecules, whereas ^13^C and ^15^N-labeled substrates allow one to trace specific fluxes, involving carbon and nitrogen transformations, respectively. Because a variety of tracers are now available, many biosynthetic fluxes can now be determined, provided that analytical tools are available to translate isotopic data into meaningful flux information. With modern and powerful mass spectrometers, metabolites can be identified based on their fragmentation signature, and stable isotope incorporation can be observed through shifts in the mass of a given fragment. The sequentially enriched species of a defined metabolite is called isotopomer. In other words, an isotopomer is a molecule with an isotopic tracer, incorporated somewhere along the molecule [25]. Isotopomers can be categorized into positional isotopomer and mass isotopomer. Whereas positional isotopomers are molecules with isotopic tracers incorporated at specific positions in the molecule of interest, mass isotopomers are those that differ in their molecular weight because of incorporation of a different number of stable isotope atoms. The conjunction of mass isotopomers detected for a given compound is called the isotopomer spectrum. In metabolic pathways with characterized atom transitions, mathematical models can use isotopomer spectrum incorporation data to reconstruct true metabolic fluxes in the given conditions. In clinical terms, stable isotope incorporation is also desirable as there is no concern about radiation. However, the main disadvantage of this technique is relatively low signal to noise ratio of isotope incorporation, which in turn necessitates relatively large amounts of labelled precursor to be administered prior to obtaining a coherent signal. Despite this disadvantage, utilization of stable isotope incorporation into molecules of interest still remains the main technique to calculate true fluxes to estimate and evaluate their significance as markers of physiology and pathophysiology *in vivo*. Especially, ^13^C metabolic flux analysis (^13^C-MFA) is able to quantify *in vivo* enzyme functions. ^13^C-MFA provides such a tool, which can be used to map the flow of carbon through entire biochemical networks [11,15,26]. Thus fluxomics, together with other omics platforms may provide insights into regulatory mechanisms underlying gene functions [21]. Details on the systems biology approach and metabolomics are discussed in several reviews [11,12,13,14,15,19,20,24,27,28,29,30,31,32,33]. In this review, we will focus mainly on the altered metabolic pathways in cancer and associated, drug resistance.

## 3. Metabolic Pathways Associated with Cancer and Drug Resistance

**Figure 2:**
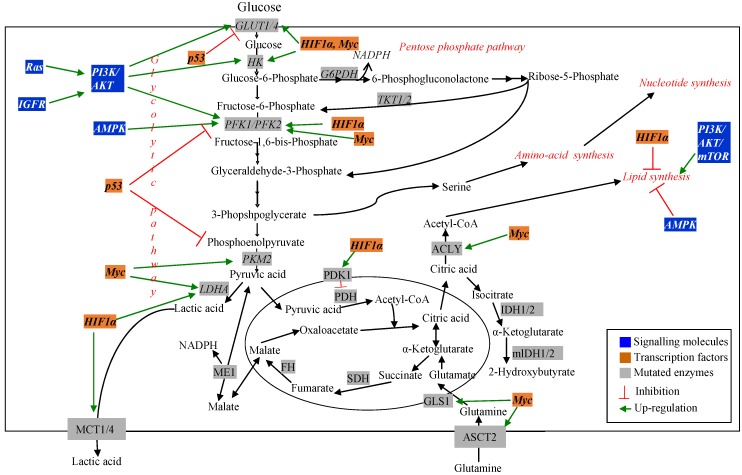
Dysregulated metabolic pathways and their regulators in cancer.

Cancer cells are highly proliferative [12]. As a result, they require continuous supply of carbohydrate, amino acid and fatty acid substrates to synthesize DNA, RNA, protein and lipid to increase biomass. Cancer cells exhibit increased rate of nutrient consumption and rerouting of metabolic processes to maintain these substrate pools and to favor *de novo* biosynthesis [11]. The presence of altered metabolism in cancer cells was first observed by using ^18^F-2-deoxyglucose positron emission tomography imaging of tumors [34]. This method is still used to determine transformed *vs.* normal cells. Since increased cellular demand exists in cancer cells, identification of altered metabolic pathways related to nutrient uptake can be therapeutic targets for cancer research [11]. Mutation in genes encoding both the regulatory molecules and metabolic pathway enzymes has been observed using different transcriptomic and proteomic techniques. MFA has been used to understand the effect of these mutations on the metabolic pathways in cancer cells [11,28]. Figure 2 schematically represents major changes in metabolic pathways and their regulators in cancer.

### 3.1. Signalling and Regulatory Molecules Associated with Metabolic Reprogramming in Cancer Cells

A number of regulatory and signaling molecules have been reported to be frequently mutated in many human cancers [35]. Mutations can be caused by point mutations, translocations, amplifications and deletions. Consequently, these, alter multiple signaling pathways, which are not observed in normal cells. These mutations affect the function of many proteins that control cell growth and death, metabolism, *etc.* and lead to cancer development. The regulatory molecules can be categorized into two main groups: (i) signaling molecules and (ii) transcription factors [20,35,36,37].

#### 3.1.1. Signaling Molecules

Signal transduction pathways define the cellular response to external stimuli, which reach the cell in the form of small molecules or chemical compounds such as hormones. These molecules bind to proteins in cell membrane and trigger activation of signaling pathways. Several signaling molecules, originating from distinct pathways have been found to be mechanistically linked to metabolic alterations in cancer cells. Many of these molecules function upstream of the metabolic pathway networks and are known to be associated with transformed metabolism in cancer cells [38].

***PI3K, AKT and mTOR*:** This axis is one of the most commonly dysregulated signaling networks in human cancer [39,40]. The phosphatidylinositol 3-kinase (PI3K) is a lipid phosphatase, which is an upstream regulator of AKT. AKT, also known as protein kinase B (PKB), is a signaling molecule and lies downstream of receptor tyrosine kinase (RTK) [41]. It is a master regulator of cellular growth and survival. AKT promotes survival by suppressing apoptosis via inhibition of the pro-apoptotic protein BAD. However, inappropriate activation of PI3K and AKT by insulin and insulin like growth factors contribute to the metabolic transformation of cancer cells. In addition, activation of oncogenes and tumor suppressor genes within this network are reported in cancer cells. PI3K/Akt pathway promotes activation of glycolytic metabolism through Akt-mediated membrane translocation of glucose transporter (GLUT1), and AKT-dependent activation of hexokinase (HK) and phosphofructokinase-2 (PFK-2). PFK2 promotes fructose-2,6-bisphosphate’s allosteric regulation of the glycolytic enzyme phosphofructokinase-1 (PFK1) [37,42,43].

AKT also stimulates *de novo* fatty acid synthesis through direct phosphorylation and activation of ACL (ATP citrate lyase). ACL catalyzes the conversion of citric acid to acetyl-CoA and OAA [37,42]. Furthermore, PI3K/AKT activates the downstream activator of cell growth regulator, “mechanistic target of rapamycin complex 1” (mTORC1). PI3K/AKT/mTOR together with the MAPK pathway has been associated with lipid biosynthesis. mTOR is also thought to stimulate *de novo* lipid synthesis. mTOR pathway plays important role in nutrient uptake, regulation of energy metabolism, and cellular survival [43]. However, mTOR is over-activated in many types of sporadic and hereditary cancers [44]. In normal cells, mTOR is one of the regulators of the autophagy pathway. Autophagy is a lysosome dependent catabolic process that occurs at the basal level in normal growing conditions to regulate the balance between protein synthesis and degradation. However, in many cancer (e.g., in breast cancer, colon cancer), autophagy supports cancer cell growth. mTOR is also activated by starvation of molecules like serine or glutamine. Higher level of mTOR was detected in renal cell carcinoma, mantle cell lymphoma, hepatocellular carcinoma, glioblastoma and breast cancer [43].

***Ras*:** Downstream of various receptor tyrosine kinases, Ras proteins are members of the GTPases and are activated by growth factors. These proteins are well known to transduce cell proliferation signals. There are different types of Ras proteins, of which K-Ras is the most frequently mutated protein in different types of cancer [38]. Ras overexpression activates several effector pathways including PI3K/AKT and MAPK, which are associated with pro-survival pathways in cancer cells. K-Ras is commonly mutated at glycine residue 12 [45]. This results in impaired GTPase activity and hyperactive signaling. In murine firbroblasts, this mutation has been reported to facilitate the decoupling of glycolytic and TCA cycle metabolism. MFA studies using ^13^C labelled tracers showed that, in murine fibroblast cells, oncogenic K-Ras decreased flux through pyruvate dehydrogenase complex (PDH), diverting most glucose-derived carbon to lactic acid [46]. The study also showed that K-Ras did not cause elevated glutamine consumption, although a greater fraction of glutamine was used for anabolic synthesis. In a different study, using non-targeted tracer fate detection (NTFD) technique, it has been shown that in human and murine pancreatic ductal adenocarcinoma (PDAC) cells, oncogenic K-Ras triggers a non-canonicals pathway of glutamine utilization. K-Ras transformed PDAC cells rely on a cytosolic glutamate-oxaloacetate transaminase 1 (GOT1). This enzyme converts glutamine-derived aspartic acid into cytosolic oxaloacetate (OAA), which is subsequently converted into pyruvate via cytosolic malic enzyme and generates NADPH that is required to maintain cellular redox balance [11]. In addition to upregulating glycolysis, K-Ras is over-expressed in many cancers and activates several effector pathways such as PI3K/AKT and MAPK pathway [26].

***AMPK*:** The AMP-activated protein kinase (AMPK) is a protein complex that plays a critical role in regulating the cellular energetic states especially under nutrient starvation condition and hypoxic condition. AMPK senses change in the cellular ratio of AMP to ATP and is activated under conditions of metabolic stress that lead to low ATP/AMP ratio. Once activated, AMPK stimulates metabolic alterations to limit energy consumption or enhance energy production, enabling the cells to adapt to a given metabolic stress [47]. AMPK can deter ATP-consuming fatty acid synthesis through an inactivating phosphorylation of acetyl-CoA carboxylase (ACC). AMPK can partially inhibit mTOR, leading to metabolic adaptation by increasing catabolism and decreasing anabolism. Furthermore, under conditions of energetic stress, one of the critical upstream activator of AMPK is liver kinase B1 (LKB1), which is recognized as tumor suppressor, shows lesion-induced deregulation of a signaling axis and metabolic control [37,48].

***ErbB*:** ErbB is a family of receptor tyrosine kinase EGFR with four receptor isoforms and more than 12 ligands. ErbB and its downstream networks are often mutated in cancers such as breast, lung and colon cancers. ErbB is also known to be over-expressed in these cancers [26]. Breast cancer patients expressing ErbB2 are treated with humanized monoclonal antibody, trastuzumab. However, trastuzumab resistance has been found to be associated with increased glucose uptake and lactic acid production in breast cancer patients [38].

***IGFR*:** Insulin like growth factor receptor (IGFR) is an important signaling molecule that functions upstream of PI3K and AKT. IGFR is reported to influence glucose uptake and cell growth in tumor cells [49].

#### 3.1.2. Transcription Factors

A number of transcription factors act as oncogenes and activates transporter proteins and enzymes of the glycolytic pathway, pentose phosphate pathway, TCA cycle and amino acid and fatty acid synthesis pathways [38]. Some of these are discussed below:

***HIF1*:** Cancer metabolism is heavily influenced by adaptation to hypoxic environment. In case of solid tumors, proliferating tumor cells surpasses the supply of nutrients and increase lactic acid production, which causes acidosis along with high levels of reactive oxygen species (ROS). In the case of solid tumors, vascularization and angiogenesis add to the overall hypoxic environment, whereas, in hematological tumors, bone marrow and lymph nodes represent hypoxic environments. As a result, tumor cells experience higher levels of stress and develop resistance to drugs because of hypoxia, acidosis and high ROS [43,50].

In the cell, the master regulator of hypoxia is hypoxia-induced factor 1(HIF-1) [51]. Upregulation of HIF-1α is associated with chemoresistance. It is a transcription factor which activates the transcription of hundreds of target genes. HIF-1 is a heterodimer and comprised of either HIF-1α or HIF-2α, and HIF-1β subunits. Of these, HIF-1α subunit or HIF-2α is regulated by oxygen level. Both the alpha (α) subunits are stabilized by hypoxia. However, the HIF-1α subunit is ubiquitously expressed, whereas the HIF-2α subunit is expressed only in endothelial, lung, renal, and hepatic cells [50,52]. Under normoxic conditions, HIF-1α undergoes proteasomal degradation through post-translational prolyl hydroxylation (PHD), mediated by von Hippel-Lindau (VHL) tumor suppressor-protein. Under hypoxic conditions, HIF-1α gets stabilized by inhibition of the prolyl hydroxylation or loss of VHL. Even metabolites like succinate or fumarate resulting from the loss of function of TCA cycle enzymes succinate dehydrogenase (SDH), or fumarate hydratase (FH) can inhibit PHD and stabilize HIF-1α [43,50].

Stabilization of HIF-1α causes transcriptional activation of genes that lead to metabolic shifts in hypoxic tumors causing higher glucose uptake and conversion of pyruvate into lactate in order to increase net ATP production by an oxygen-independent mechanism. HIF-1 induces the expression of several genes associated with fermentative glucose metabolism, which includes glucose transporters, glycolytic enzymes, lactate dehydrogenase A (LDHA), and pyruvate dehydrogenase kinase-1 (PDK1) [37]. Activation of PDK1 by HIF1 diverts pyruvate away from mitochondrial oxidation and 6-phopshpfructo-2-kinase/fructose-2,6-bisphosphatase 4 (PFKFB4), which degrades fructose-2,6 bis-phosphate (F2,6BP). F2,6BP is a powerful allosteric activator of phosphofructokinase 1 (PFK1) and converts fructose 1-phosphate to fructose 1,6-bisphosphate (F1,6BP). While this is a rate limiting step in glycolysis, increased PFKFB4 is observed in prostate cancer cell lines and diminishes PFK1 activity [37]. This also diverts glucose into PP pathway, elevating NADPH level to titrate ROS. Activation of LDHA is directly involved in diverting pyruvate flux to lactate and PDK1 is indirectly involved in negative regulation of entry of pyruvate into the mitochondria [43,50].

In acute lymphoblastic leukemia (ALL), levels of HIF-1α, GLUT1, GLUT3, CA4, and glyceraldehyde-3-phosphate dehydrogenase (GAPDH) were significantly greater in leukemic cells compared to healthy blood cells. Moreover, leukemias with higher glycolytic rates showed resistance to chemotherapeutics, e.g., glucocorticoids. Elevated glucose uptake rate and redirection of glycolytic pyruvate to lactate was also observed in renal cell carcinoma (RCC) under hypoxic conditions [43,53].

Reactive oxygen species (ROS) comprised of superoxides (O_2_^−^), hydroxyl radicals (OH^−^) and hydrogen peroxide (H_2_O_2_) is produced from mitochondria and damaged membranes, which are mutagenic [54,55]. Also, the free radical gas nitric oxide, which is endogenously produced in mammalian cells acting as a signaling molecule, has been reported to be involved in different cancers [56]. Superoxide dismutases (SODs) are essential to maintain the redox homeostasis through the conversion of superoxide to hydrogen peroxide and neutralized by catalases to water and oxygen. However, cancer cells have the ability to handle ROS, and increased ROS is reported to modify a critical sulfohydral group of pyruvate kinase M2 (PKM2). This inactivates PKM2, resulting in the shunting of glucose away from glycolysis toward the PP pathway. Since NADPH is generated at PP pathway, this altered flux of glucose toward PP pathway plays essential role in redox homeostasis in cancer cells. ROS also stabilizes HIF-1 and the consequence of its stabilization has already been discussed. ROS plays a role in intracellular signaling through alterations of the oxidative status of regulatory protein sulfhydryl moieties. The antioxidant properties of cancer cells can profoundly influence metabolic stress and increased resistance to certain therapeutics [55,57].

***Myc*:** c-Myc is a transcription factor and regulates cell growth, proliferation and activation of downstream growth factor-mediated signaling. However, in several tumors, c-Myc is a proto-oncogene, where it stimulates enhanced expression of many genes involved in energy production and biomolecule synthesis, which are required for rapid proliferation. Myc promotes aerobic glycolysis by enhancing the expression of GLUT1 and LDHA [58]. c-Myc enhances the glycolytic pathway by increasing the expression of glucose transporters GLUT1 through pyruvate kinase as well as lactate dehydrogenase A (LDHA), thereby allowing glucose derived carbon as lactate. Myc also upregulates amino acid metabolism pathways especially glutamine utilization, glutamine transporters and genes involved in both mitochondrial biogenesis and glutaminolysis. Myc also induces the expression of serine/glycine metabolism and fatty acid synthase (FAS) enzyme, which is involved in lipid biosynthesis. c-Myc works in concert with HIF-I alpha to confer metabolic advantages [50].

c-Myc is also activated independent of growth factor stimulation. Metabolic flux analysis using ^13^C glucose and ^13^C glutamine in P493-6-B-cells showed that Myc over-expression increased mitochondrial fluxes 3-4 fold and more pronounced impact on amino acid metabolism was observed. These cells have been shown to grow in the total absence of glucose by relying on complete oxidation of glutamine to generate ATP [11]. Myc also regulates glutaminolysis where glutamine consumption exceeds the cellular requirement for protein and nucleotide biosynthesis. c-Myc induces the expression of nucleotide metabolism pathway enzymes inosine 5′-monophosphate dehydrogenase, serine hydroxymethyltransferase, adenosine kinase, and adenylate kinase 2 [43].

Myc regulated proline metabolism via proline oxidase was observed in Myc-inducible human Burkitt lymphoma cell lines and in human prostate cancer cells lines. Metabolic flux analysis shows that Myc induced glutaminolysis persists in hypoxia in Burkitt lymphoma cell lines. In these cells, the TCA cycle is solely supported by glutamine. ^13^C pyruvate imaging in mice revealed that the progression of Myc-driven liver cancer is modulated via LDHA. However, alanine metabolism was found to be associated with both tumor formation and regression [11,43].

***P53*:** p53 is a tumor suppressor and is encoded by the TP53 gene [59]. It is a transcription factor, which regulates the expression of more than 300 different promoter elements and is one of the most vital defenders in cellular response to stresses. p53 plays important role in DNA damage repair, regulating the downstream cell cycle regulator p21, activating caspases via cleavage of pro-caspase 3, 7 and 9, regulating expression of PARP1 (poly-ADP-ribose polymerase 1) and even in regulating autophagy pathway proteins DRAM at the transcriptional level. PARP1 is involved in differentiation, proliferation, tumor transformation and in repairing of single stranded DNA damage. P53 is also associated with the regulation of metabolic pathways [36]. In normal cells, p53 inhibits glycolysis and upregulates the expression of TP53-induced glycolysis and apoptosis regulator (TIGAR), causing decreased level of fructose-2,6-bisphosphate and decreased glycolytic rate. P53 transcriptionally induces the synthesis of cytochrome oxidase 2 (SCO2) and TP-53 induced glycolysis and apoptosis regulator (TIGAR), but represses the expression of various glucose transporters (GLUTs), the glycolytic enzyme phosphoglycerate mutase and pyruvate dehydrogenase kinase 2 (PDFK2) in normal cells. However, p53 is one of the most commonly mutated genes in cancer and increases glycolysis in cancer cells [36,43,44,60].

A resistant phenotype of p53 mutation induced by ionizing radiation (IR) showed higher expression of PARP1 level. Furthermore, inactivation of p53 in tumor cells shifts metabolism from mitochondrial respiration towards glycolysis including overexpression of glucose trasporters GLUT1, GLUT4 and GLUT3, enhancement of the expression of glycolytic enzymes such as phosphofructokinase and phosphoglycerate mutase, suppression of mitochondrial respiration by inhibiting the synthesis of cytochrome c oxidase 2 and activation of AKT and HIF, which are effectors of downstream of PI3K [36,37]. Inactivation of p53 in tumor cells also accelerates glycolysis and increases metabolic flux into the pentose phosphate pathway (PPP). This is mediated by two different mechanisms: firstly, p53 binds glucose-6-phosphate dehydrogenase (G6PDH), which is the rate-limiting enzyme of PPP, inducing a conformational conversion of G6PDH, which inhibits PPP. Tumor-associated p53 mutants lack the G6PDH-inhibitory activity and thereby do not inhibit PPP and associated NADPH production. NADPH contributes to the cellular defense against oxidative stress and is required for fatty acid synthesis; secondly, TIGAR is a negative regulator of the glycolytic enzyme phosphofructokinase-1 (PFK1). PFK is a glycolytic pathway enzyme which converts fructose-6-phosphate to fructose-1,6-bisphosphate. TIGAR reduces the levels of fructose-1,6-bisphosphate and thereby blocks glycolysis at this step, thus driving glucose flux through the oxidative PPP for production of ROS-titrating NADPH antioxidant [43]. In addition, p53 promotes glutaminolysis by activating the expression of glutaminase 2 (GLS2). This increases the level of glutathione, a key antioxidant [36,43].

***SREBP*:** SREBP-1 is a member of the SREBP family of transcription factor, which induces the expression of several genes that are involved in fatty acid and sterol biosynthesis in response to growth factors or intracellular sterol levels. Recent studies showed that SREBP-1 is also a downstream effector of mTORC1. Hyperactivation of mTORC1 leads to deregulation of *de novo* lipid synthesis for sustained membrane production and cell proliferation [37].

### 3.2. Metabolic Pathway Genes Associated with Altered Metabolism in Cancer Cells

Proliferating cancer cells must accumulate biomass, replicate DNA and divide. As a result, they require continuous supply of nutrient and energy. To meet this demand, mutation in several metabolic pathway enzymes were observed. These are discussed below:

#### 3.2.1. Glucose Metabolism Pathways in Cancer Cells

Glucose serves as the major energy and nutrient source for proliferating cells. In normal cells, glucose is metabolized in the presence of oxygen via the mitochondrial oxidative phosphorylation (PXPHOS) pathway to generate ATP [61]. In these processes, 34 molecules of net ATP are produced from complete oxidation of one glucose molecule. In contrast, cancer cells use the glycolytic pathway to generate energy and produce lactic acid even in the presence of oxygen. Initially it was hypothesized that cancer cells have defective mitochondria. However, using gene knockout techniques, with siRNA against lactate dehydrogenase (LDHA) and chemical inhibitors of pyruvate dehydrogenase kinase (PDK), reduced cancer cell proliferation in presence of functional OXPHOS was observed [62,63]. Other studies showed that, glycolytic metabolism helps cancer cells to grow at a higher rate in the presence of unlimited supply of glucose. Glucose also serves as carbon skeleton for amino acid synthesis, nucleotide synthesis and fatty acid synthesis in cancer cells, which also leads to increased biomass [64]. In leukemia and multiple myeloma (MM), increased glycolysis and ATP levels is associated with drug resistance via enhanced drug efflux activity [65]. Furthermore, glucocorticoid resistance in childhood ALL is also associated with increased glycolysis [66]. Dysregulation of several enzymes associated with glucose metabolism has been reported in different types of cancer [63,67,68,69].

#### 3.2.2. Glucose Transporters

Mammalian cells obtain glucose by glucose utilizing transporters (GLUT). It is the first rate limiting step in glucose metabolism. These proteins help glucose to be transported across the plasma membrane. The human GLUT family consists of 14 proteins. However, a number of GLUT proteins are reported to be dysregulated or over-expressed in malignant cells. For example, GLUT4 is reported to be over-activated in multiple myeloma (MM) cells, which correspond well with high basal glucose consumption in these cells. Another GLUT protein, GLUT3 was found to be up-regulated in glioblastoma patients and it was also associated with resistance to temozolomide, a drug which is used to treat these patients in combination with radiotherapy [70].

#### 3.2.3. Glycolytic Pathway

***Hexokinase (HK)*:** Hexokinase (HK) is the first enzyme of the glycolytic pathway. It controls phosphorylation of glucose into glucose-6-phosphate (G6P). G6P is an important intermediate for both the glycolytic pathway and the PP pathway. Increased HK activity has been detected in human breast cancer cells and in leukemia and multiple myeloma (MM). HK showed more than 50% activity in mitochondrial fraction. Studies also showed that growth rate of cancer cells is directly proportional to specific activity of HK [70,71,72].

In mammalian system, HK exists in two different molecular forms, which are soluble and particulate forms. The soluble form is less active and sensitive to feedback inhibition by glucose-6-phosphate. On the other hand, the particulate form is more active and sensitive to feedback regulation. The HK bound to mitochondrial membrane resembles particulate form and is less sensitive to inhibition by glucose-6-phosphate. This form of HK is also known as hexokinase-2 (HK2) and has been found to be preferentially expressed in various cancers. HK2 interaction with mitochondrial membrane protects it from proteolytic degradation. Investigations are currently undergoing to check the efficacy of HK inhibitors [70,73].

***Phosphofructokinase (PFK)*:** Phosphofructokinase-1 (PFK1) is a rate limiting enzyme in glycolysis, which senses the cellular energy levels. PFK1 catalyzes the forward reaction by adding a second phosphate group to fructose-6-phosphate (F6P) and produces fructose-1,6-bisphosphate using an ATP molecule [74]. The reverse reaction is carried out by bisphosphatase (BP1). PFK1 is allosterically regulated by fructose-2,6-bisphosphate (F2,6BP), ATP, ADP and AMP. ATP allosterically deactivates PFK1, while F2,6BP, ADP and AMP activates PFK. PFK activity is overexpressed in many tumor cells, and Myc oncogene also upregulates the expression of PFK1. In addition, citrate, a TCA cycle metabolite is also an inhibitor of PFK [70].

Liver-type PFK is preferentially expressed in many cancer cells and they show different sensitivity towards allosteric regulators, compared to normal cells. PFK from human glioma cells are less sensitive to inhibition by other metabolites, such as citrate, but highly sensitive to activation by fructose-2,6-bisphosphate [75]. Since PFK plays an important role in regulating glycolytic flux, it is an important target of cancer treatment [70].

***Pyruvate kinase (PK)*:** Pyruvate kinase (PK) is the rate-limiting enzyme that catalyzes the conversion of phosphoenolpyruvate (PEP) to pyruvate and produces ATP. The mammalian PK exists in four isoforms: the muscle forms M1 and M2, the liver form L and the red blood cell form R, and are present in different cell types. Of these, PKM1 is constitutively active, tetrameric form of PK and is found in normal adult cells. On the other hand, PKM2 is the embryonic and tumor isoform, and forms both, tetramers and less active dimers. PKM2, has been found to be associated with poor response to oxaliplatin in colorectal cancer. It is also associated with poor response to 5-FU, one of the most commonly used chemotherapy to treat cancer [36,70].

The active tetramer form of PKM2 requires fructose-1,6-bisphosphate and this tetrameric form is found in differentiated tissues and normally proliferating cells [76]. PKM2 has a high affinity to its substrate PEP and promotes the conversion of PEP to pyruvate. The less active form PKM2, which is found in its less active dimeric form, causes all glycolytic intermediates above PK to accumulate in cancer cells. Studies revealed that phosphorylation of tyrosine at 105 site of PKM2 releases fructose-1,6-bisphosphate from PKM2 to the less active dimeric form and reduces PKM2 activity. In addition, high intracellular concentration of reactive oxygen species causes oxidation of the cysteine 358 residue in PKM2 and inhibits the multimer formation, which eventually lowers the activity of PKM2. The suppression of PKM2 leads to the accumulation of all of the glycolytic intermediates upstream of PK, the most important of which is PEP, which is the substrate for PKM2, and inhibits the glycolytic enzyme triose phosphate isomerase (TPI). TPI converts the three carbon sugars glyceraldehyde 3 phosphate and dihydroxyacetone phosphate, leading to the activation of pentose phosphate pathway. Increased activity of the PP pathway produces increased ribose-5-phosphate and NADPH. The former is used for nucleic acid synthesis and the latter is used to maintain the reducing environment, required for antioxidant enzyme activity, and for recycling the antioxidant glutathione. The changes in PKM2 activity in cancer cells produce antioxidant glutathione, protecting cancer cells against reactive oxygen species [36,70].

***Pyruvate dehydrogenase kinase (PDK) and lactate dehydrogenase A (LDHA)*:** Pyruvate dehydrogenase (PDH) converts pyruvate to acetyl-CoA. PDH is the rate limiting enzyme in this step. However, under anaerobic condition, pyruvate is redirected into lactic acid production, where the ratio of cytosolic NADH/NAD^+^ increases due to decrease of NADH oxidation by the mitochondria. Two enzymes of the TCA cycle are involved in the increased production of lactic acid: pyruvate dehydrogenase kinase (PDK) and lactate dehydrogenase A (LDHA). Of these, four isotypes of PDK exists. Under hypoxic condition, PDK1 inhibits PDH, which in turn inhibits the conversion of pyruvate to acetyl-CoA, shunting pyruvate to lactate. On the other hand, the PDK3 is less sensitive to high concentrations of pyruvate and its expression is upregulated by HIF-1alpha as it binds to the promoter of PDK3, thus keeping increased production of pyruvate to lactate [55,77]. PDK3 is associated with chemoresistance to cisplatin, paclitaxel or oxaliplatin. It also contributes to hypoxia induced drug resistance in cervical and colon cancer [70].

The other enzyme, lactate dehydrogenase (LDH) is crucial to maintain the reducing environment and energy source in the form of NADH. LDHA is associated with paclitaxel or trastuzumab resistance in breast cancer [65]. In the absence of mitochondrial function, regeneration of NAD^+^ from the conversion of pyruvate to lactate is catalyzed by LDH. LDH exists in several isoforms. Over-expression of LDH5 has been reported in several cancer types such as non-small-cell lung cancer, colorectal cancer, gliomas, *etc.* and increased lactic acid production has been reported in certain neoplasms [70]. LDH5 is important in promoting anaerobic glycolysis and is transcriptionally regulated by HIF-1α and HIF-2α. Under hypoxic condition, regeneration of NAD^+^ for glycolysis is solely dependent on LDH enzymes. Lactic acid also lowers the intracellular pH in tumor environment, and the acidic pH protects the cancer cells from the surrounding leukocytes that tend to destroy the cancer cells. Lactic acid also provides physiological barriers and thus contributes to drug resistance. Targeting LDH may therefore provide multiple advantages in cancer treatment [70].

#### 3.2.4. Pentose Phosphate Pathway

The pentose phosphate pathway (PPP) is another route of glucose metabolism in cell. Whereas glycolytic pathway of glucose metabolism generates ATP, the products of the pentose phosphate pathway are mostly associated with biosynthetic pathways. For example, ribose and NADPH are synthesized in this pathway, which are required for nucleic acid biosynthesis and maintenance of reductive environment. PP pathway is further divided into two branches: oxidative pathway and non-oxidative pathway. NADPH is synthesized in the oxidative pathway and ribose is synthesized in the non-oxidative pathway. Mutations in the genes encoding enzymes of both pathways are reported in cancer cells [70].

***Glucose-6-phosphate dehydrogenase (G6PDH)*:** Glucose-6-phosphate dehydrogenase (G6PDH) is the rate limiting enzyme of the oxidative branch of the PP pathway. G6PDH is involved in the maintenance of glutathione (GSH) level in the cell. GSH is required to maintain reducing environment in the cell, which is dependent on the NADPH generated by the reaction G6PDH performs in conversion of glucose 6 phosphate (G6P) into 6-phospho-gluconate (6PG). In melanoma cells and in PC-12 neural cells, over-expression of G6PDH showed significant resistance to apoptosis. This is correlated with the anti-apoptotic role and pro-survival role of G6PDH in cancer cells. In addition, breast cancer cells labelled with 1,2-^13^C glucose showed a significant increase in oxidative PPP flux. The study also showed glutamine derived carbon which is indicative of reverse glycolytic flux to generate reducing power in the oxidative branch of the PPP [16,78].

***Transketolase (TK)*:** Transketolase (TK) is an enzyme of the non-oxidative branch of the PP pathway [78]. It has two isoforms, TKT1 and TKT2. Of these, the TKT1 isoform is upregulated in several tumor types, and ribose-5-P (R5P) synthesized from the catalytic action of TKT1 is high in certain tumor cells. In addition to this enzyme, sedoheptulose 7-phosphate (S7P), which is an intermediate of the non-oxidative branch of PPP, participate in highly reversible non-oxidative reactions that involve R5P, GAP or erythrose 4-phosphate (E4P) and F6P. Of these, R5P is the constituent of nucleic acid, synthesized by the *de novo* pathway. In fact, cancer cells almost exclusively use this pathway for nucleotide synthesis. Since cancer cells depend on PP pathway for higher nucleotide synthesis and NADPH generation for reductive biosynthesis, drugs or molecules that can decrease the flux through PP pathway can be used to overcome resistance to chemotherapy [78].

#### 3.2.5. Tri-Carboxylic Acid (TCA) Cycle

In addition to aberrant changes in the glycolytic pathway flux, mutation in genes encoding certain TCA cycle enzymes and differential metabolic flux have been reported in different types of cancer. Metabolic flux analysis using NMR or mass spectrometry mapping showed altered metabolic flux in cancer cells by tracing the incorporation of ^13^C labeled metabolites arising from ^13^C glucose or ^13^C glutamine treated cells [28,79].

***Iso-citrate dehydrogenase (IDH)*:** Iso-citrate dehydrogenase (IDH) is an enzyme of the TCA cycle, which catalyzes the conversion of isocitrate to α-ketoglutarate with concomitant reduction of NADP^+^ to NADPH. IDH exists in two isoforms: IDH1 and IDH2. Both forms catalyze the oxidative decarboxylation of isocitrate to α-ketoglutarate (α-KG). A genome wide analysis, consisting of sequencing protein-coding genes and RNA sequencing of low-grade glioma, secondary glioblastoma, and acute myeloid leukemia in humans, revealed mutations in the active site of the enzyme IDH1 and IDH, in particular, heterozygous somatic mutations at arginine R132 of IDH1 and at R140 or R172 of IDH2 [28,80,81,82].To understand the effect of these mutations on the cellular metabolome, U87MG glioblastoma cells were stably transfected with myc-tagged wild type IDH1 and R132 mutant IDH1 and compared the metabolomes of these cells using an untargeted liquid chromatography/mass spectrometry (LC/MS)-based untargeted metabolomics profiling approach. This provided un-biased comparison of metabolites, which were altered between the two groups. The result showed that R132 mutation in IDH1 generated the oncometabolite 2-hydroxyglutarate (2-HG). The same study also showed that whereas the wild-type IDH1 produced α-KG and NADPH, IDH1 mutant consumed NADPH and reduced α-KG to 2-HG. These studies also revealed the loss of function of a mutated enzyme [28,70,80].

Further metabolic profiling by another researcher group showed that mutation in IDH1 and with the treatment of 2-HG resulted metabolic changes in the downstream metabolites including amino acids, GSH metabolites, choline derivatives and TCA cycle intermediates. This group used targeted LC-MS/MS and found that N-acetylated amino acids NAA and NAAG were significantly low in cells containing mutated IDH1. IDH1 derived 2-HG has also been shown to increase CpG island methylation [28,83].

Several other research group using ^13^C labeled carbons arising from ^13^C-glucose or ^13^C glutamine showed that oncometabolite 2-HG also competitively inhibits several α-KG dependent dioxygenase enzymes that use α-KG as a substrate to catalyze a wide range of reactions including alterations in histone and DNA methylation, biosynthesis of collagen or L-carnitine and responses to hypoxia. Mutations in IDH1 or IDH2 increase 2-HG, which is a PDH2 inhibitor. This suppresses the activity of PDH and stabilizes HIF-1α, which eventually activates glycolysis [50]. Reductive carboxylation of α-KG in aggressive cancers showed that isocitrate isomerizes to citrate and generates acetyl-COA (ACOA). This is used for cytosolic fatty acid synthesis. Moreover, knocking down IDH2 impaired cell proliferation in cancer cells [28].

***Succinate dehydrogenase (SDH) and fumarate hydratase (FH)*:** Succinate dehydrogenase (SDH) and fumarate hydratase (FH) are two enzymes of the TCA cycle, where the former one catalyzes the conversion of succinate to fumarate and the later one catalyzes the conversion of fumarate to malate, respectively. Dysfunction in these two enzymes has been linked to the development of various cancers [84]. For example, germline mutation in fumarate hydratases (FH) is reported high in familial cancer syndromes, and in skin, renal and uterus cancers. Germline inactivating mutations in SDH gene has been linked to paraganglioma. Mutations in SDH and FH enzymes result in the accumulation of fumarate and succinate, respectively. These metabolites are oncogenic as the accumulated metabolites can pass through the inner mitochondrial membrane and enter the cytosol. These then inhibit dioxygenases and prolyl hydrolases, the enzymes that are involved in the degradation of HIF-1α under normoxic condition. The elevation of HIF-1α is pro-oncogenic and lead to cancer development. In addition, loss of SDH and FH function leads to pseudo hypoxic environment, even in the presence of oxygen, which is linked to cancer development [70,77]. High levels of fumarate and succinate has also been reported in certain cancers such as colon, lung and prostrate tumor tissues [70,77].

#### 3.2.6. Amino Acid Metabolism Pathways

Amino acid metabolism pathways involve one carbon metabolism and glutaminolysis. Like glucose, glutamine serves as nutrient and energy source in cancer cells. However, few other amino acids are also used by cancer cells, and differential expression of certain amino acid transporters and enzymes involved in catabolizing the amino acids are observed in cancer cells [70].

***Amino acid transporters*:** Studies showed that the plasma membrane expresses a multitude of amino acid transporters, also known as solute carriers (SLC) for transport of amino acids across the membrane. In human, there are approximately 400 SLCs. Of these, expression of SLC6A14 is upregulated in tumors of epithelial origin which includes colon cancer, cervical cancer, breast cancer and pancreatic cancer [55]. Cell internalization of amino acids is rate-limiting and may involve different amino acid transporters. For example, cell internalization of L-glutamine and its quick efflux depends on the presence of essential amino acids and involves the bi-directional transporter SLC7A5/SLC3A2. The other amino acid transporter, SLC1A5 regulates glutamine uptake, loss of which is found to inhibit cell growth and activate autophagy [70].

***Glutaminolysis*:** Glutamine is an essential amino acid, and like glucose, glutamine is also metabolized by TCA cycle. However, it differs from glucose metabolism in that, while glucose metabolism is affected by hypoxia, glutamine metabolism is not affected by the presence or absence of oxygen. Instead, mass spectrometry and NMR-based metabolomics studies have provided valuable insights into how cancer cells undergo metabolic switch that consists of glutamine dependent anaplerotic pathways that contributes to citric acid and lipid metabolism through the reversal of the TCA cycle or reductive carboxylation of α-KG by isocitrate dehydrogenase (IDH). Untargeted and unbiased metabolomics approaches have also uncovered neomorphic roles associated with mutant IDH, which is associated with the generation of the oncometabolite 2-HG. Subsequent studies showed that this 2-HG acts as a demethylase inhibitor, leading to epigenetic changes that may drive cancer [28,55].

The entry of glutamine into the TCA cycle is carried out by two enzymes, glutaminase (GLS) and glutamate dehydrogenase (GDH). GLS converts glutamine into glutamate and GDH converts glutamate into α-KG. Glutamine is the most abundant amino acid in plasma. However, in the plasma of cancer patients, glutamine level is low as tumor cells use it for energy generation and biosynthetic purposes. In addition, elevated levels of basal GLS activity was found in transformed fibroblasts and breast cancer cells. GLS is often upregulated in MYC transformed cells as well [55]. Cancer cells with elevated glutamine synthetase do not require exogenous glutamine. Transport of essential amino acids like glutamine across the cell membrane is the rate-limiting step in glutamine metabolism and regulates mTOR activation, where mTOR is a regulator of autophagy. Interestingly, cells cultured in glutamine containing medium showed increased autophagy, which was neither due to nutrient depletion nor inhibition of mTOR [70,85].

***Other amino acids*:** Other than glutamine, recent experimental approaches showed that other amino acid metabolic pathways are also dysregulated in cancer and contribute to cancer aggressiveness. By using nuclear magnetic resonance (NMR)-based, two-dimensional heteronuclear single-quantum correlation spectroscopy (HSQC), to quantify steady state levels of glucose-derived metabolites in HEK293T cell lines, following labelling with ^13^C glucose, large amount of glycolytic carbon diverted into serine and glycine. Another study using targeted LC-MS/MS based measurements of metabolites showed that substantial portion of ^13^C glucose was diverted from 3-phosphoglycertate to 3-phosphoenolpyruvate by the enzyme phosphoglycerate dehydrogenase (PHGDH). It was also observed that serine synthesis pathway is responsible for approximately 50% of the conversion of glutamine to α-KG [28,86]. Low asparagine level was detected in the plasma of childhood acute lymphoblastic leukemia. Decreased activity of asparagine synthesizing enzyme (ASNS) was observed in these patients [77].

Glycine, an essential amino acid and a precursor for *de novo* purine formation is upregulated by elevated level of HIF-1α, which is pro-oncogenic [77]. The level of glycine decreases rapidly with the disruption of the HIF-1 signaling. In addition, changes in glycine synthesizing enzymes are found in breast cancer patients. For example, glycine is produced from serine by serine hydroxymethyl transferase (SHMT), and the reaction is mediated by the cofactor tetrahydrofolate (THF) which is generated by methylene tetrahydrofolate dehydrogenase (MTHFDH). High expression levels of these enzymes were observed in breast cancer patients and in the mitochondrial forms of the enzymes SHMT2, MTHFD2 and MTHFD1L, but not in their cytosolic form. Glycine was rapidly consumed by fast-growing melanoma cells. Moreover, activation of the glycine decarboxylase complex or glycine cleavage system (GCS) was reported to play important role in promoting tumorigenesis is non-small cell lung cancer [77,87]. While low levels of glutamine and asparagine is observed in cancer patients, alanine and glycine levels are upregulated in different types of cancer. For example, high level of alanine is detected in hepatoma and brain tumors, gliomas, meningiomas and dysembryoplastic neuroepithelial tumors. Alanine in conjunction with lactic acid increases in tissues during hypoxia [77].

#### 3.2.7. Lipid Metabolism Pathways

Cancer cells require fatty acids for the synthesis of membranes as well as for the generation of lipid signaling molecules to trigger cell proliferation leading to malignancy. Several metabolites of the lipid metabolism pathways are detected at an elevated level in cancer cells. Citric acid from TCA cycle serves as the precursor for endogenous fatty acid synthesis. Citric acid is exported to the mitochondria and converted to malonyl-CoA by the action of the enzymes ATP citrate lyase (ACYL) and acetyl-CoA-carboxylase (ACC). Malonyl-CoA acts as the substrate for the fatty acid synthesis enzyme fatty acid synthase (FASN), which is a multifunctional protein, and converts malonyl-CoA to palmitate [66]. Although FASN level is low in normal tissues, its expression level is higher in several tumor and cancer cells (e.g., colorectal cancer and endometrial cancers). FASN has been linked to ErbB2-induced breast cancer chemoresistance to docetaxel. FASN is also associated with resistance to trastuzumab or adriamycin resistance in breast cancer. In pancreatic cancer, FASN is reported to contribute to resistance to gemcitabine or radiation therapy [36,88].

## 4. Anticancer Agents that Target Metabolic Pathways and Their Regulators

Conventional chemotherapy drugs used in cancer treatment include: (i) alkylating agents that directly damage DNA to prevent the cell from reproducing; (ii) antimetabolites that interfere with DNA and RNA synthesis by substituting for the normal building blocks of RNA and DNA; or (iii) enzyme inhibitors that affects DNA replication and cell cycle. The first chemotherapy drug used to treat cancer is an antimetabolite, 5-fluorouracil (5FU), a pyrimidine analog, which irreversibly inhibits thymidylate synthase, resulting in the depletion of thymidine triphosphate (TTP), one of the four nucleotide triphosphates used in DNA synthesis, *in vivo*. A common problem with these drugs is that they are non-specific and deleterious to normally proliferating cells. As a result, use of these drugs is commonly associated with non-reversible side-effects and associated health complications [89]. As opposed to traditional chemotherapy, targeted cancer therapies include drugs or other substances that inhibit the growth of cancer cells by interfering with specific molecular targets that are involved in tumor development and metastasis. Targeted therapies are currently the focus of anticancer research and find itself at the heart of the concept of personalized medicine. Till date, a large number of targeted, cancer drugs have been approved by the Food and Drug Administration (FDA) to use in specific types of cancer [89].

It has been recognized for many years that cancer cells have high demand for metabolic inputs to support their abnormal rate of proliferation. In order to meet their excess demand for energy, cancer cells depend a lot on altered metabolic pathways such as aerobic glycolysis, fatty acid synthesis and glutamine metabolism. Furthermore, emerging evidence now suggests that these dysregulated metabolic features are also linked to therapeutic resistance in cancer treatment. Targeting cancer metabolism has therefore emerged as a promising new strategy for the development of selective anticancer agents in recent decades. Some of the recently developed drugs that target the upstream signaling proteins or transcription factors linked to cancer metabolism and others that directly target the metabolic pathway enzymes are shown in Table 1. While a few of these drugs are FDA approved, a number of them are either at the clinical or pre-clinical stages of development [42,90].

**Table 1:** Drugs/compounds targeting different proteins/enzymes of the metabolic pathways [42,65].

Pathways	Target Proteins/Enzymes or Metabolites	Drugs/Compounds	Cancer/Tumor Type	Clinical Trial Status
**Signalling proteins and transcription factors**				
	mTORC1	Temsirolimus and Everolimus	Metastatic/non-metastatic solid tumors	US FDA approved
		Ridaforolimus and other rapalogues	Pancreatic, endometrial and glioblastoma; lymphoma.	Phase I/II
	mTORC1 and mTORC2	Torin1 and PP242	-	Preclinical
	HIF1α	PX-478	Advanced solid tumor and lymphoma	Phase I
		Acriflavine	-	Preclinical
	Hypoxia	Tirapazamine and other bioreductive compounds	Cervical, SCLC, NSCLC	Phase III
	Hypoxia, VEGF and VEGFR	Bevacizumab	Malignant glioma, NSCLC, ovarian and colorectal	US FDA approved
	IGF1R	Dalotuzumab (MK-0646), BIIB022, AVE1642 *etc*.	Solid tumors of NSCLC, pancreatic, hepatocellular carcinoma (HCC) and metastatic breast cancer	Phase I/II
	PI3K and mTOR	BEZ235, XL765, SF1126 and BGT226	Malignant glioma and NSCLC	Phase I/II
	PI3K	GDC-0941 and PX866	Metastatic breast cancer and non-Hodgkin’s lymphoma	Phase I
	AKT	Perifosine and GSK690693	Renal cancer, NSCLC and lymphoma	Phase I/II
	AMPK and Complex I (mitochondrial)	Metformin	Solid tumors and lymphoma	US FDA approved
**Metabolic pathway enzymes**				
Nucleotide biosynthesis pathway	DNA and RNA synthesis	5-FU, cytarabine and methotrexate	Different types of tumors	US FDA approved
	DNA synthesis	Folate, choline, methionine,		Lab studies
	Methyltransferases	Betaine, selected B vitamins, Flavonoids, EGCG, genistein		Lab studies
	Histone deacetylases (HDAC)	Butyrate, sulforaphane, Allylmercaptan, 3,3-Diindolylmethane		Lab studies
	Histone acetyltranferase	Anacardic acid, garcinol, Curcumin, EGCG, Genistein		
	Acetylation of non-histone proteins	Butyrate, cambinol, Dihydrocoumarin, genistein		
Glycolysis pathway	GLUT1	Phloretin	Colon cancer and leukemia	-
	GLUT1	WZB117	Lung cancer and breast cancer	-
	GLUT4	Ritonavir	Multiple myeloma	-
	Hexokinase	2-deoxyglucose (2-DG)	Leukemia, cervical cancer, hepatocarcinoma, breast cancer, small lung cancer, lymphoma and prostate cancer	PhaseI/II
	Hexokinase Hexokinase	Lonidamine (LND)	Benign prostatic hyperplasia, leukemia and lymphoma	Phase III
		3-bromopyruvate (3-BrPA)	Leukemia, multiple myeloma, colon cancer and leukemia	Preclinical
	Pyruvate kinase M2 (PKM2)	shRNA	Lung cancer	-
	Pyruvate kinase (PK)	TLN-232	Metastatic melanoma and renal cell carcinoma	Phase II
	Lactate dehydrogenase (LDHA)	Oxamate	Breast cancer	
Pentose phosphate pathway (PPP)	Glucose-6-phosphate dehydrogenase (G6PDH)	Resveratrol	Colon cancer	
	Transketolase (TK)	Oxythiamine (OT)	Colon cancer	
	G6PDH and TK	Avemar	Jurkat T cells (Leukemia)	
	G6PDH, 6PGDH and Transaldolase TA	Combination of arginine and ascorbic acid	Human hepatoma cell lines (HA2T/VGH)	
	G6PDH, also depletion of ribose-5-phosphate (R-5P)	Dehydroepiandrosterone (DHEA)	Indirect study on polycystic ovary syndrome	
TCA cycle	Pyruvate dehydrogenase kinase (PDK3)	siRNA	Cervical cancer and breast cancer	
	Pyruvate dehydrogenase kinase (PDK1)	Dichloroacetate (DCA)	Fibrosarcoma, colon cnacer, lung cancer, squamous cell carcinoma and prostate cancer	
Fatty acid synthesis	FASN	Cerulenin and C75	Breast cancer	
		Orlistat	Breast and pancreatic cancer	Preclinical
	ATP-citrate lyase	SB-204990	-	Preclinical
Amino acid metabolism pathway	Glutamine	Phenylacetate	Brain tumors	Phase II
	Asparagine	Asparaginase and pegasparaginase	Acute lymphoblastic leukaemia (ALL), T-cell lymphoma (TCL) and B-cell lymphoma (BCL)	Phase II/III
	Arginine	Arginine deiminase	Metastatic melanoma and hepatocellular carcinoma	Phase I/II

Metabolic rearrangements in cancer have been shown to be linked to the activation of proto-oncogenes and to the inactivation of tumor suppressor genes, reinforcing the notion that intermediate metabolism and signal transduction are highly correlated. Major upstream regulators of metabolic pathways that are important targets for anticancer drugs include HIF, PI3K, AKT, mTOR and AMPK. Targeting HIF prevents metabolic shift or adaptation of tumor cells to hypoxia and anticancer agents such as PX-478 that reduces HIF-α level *in vitro* and *in vivo* was found to show potent anti-tumor effects. Another drug, acriflavine, which blocks the dimerization of HIF subunits reduced tumor growth and vascularization in mice bearing prostate cancer xenografts [42,91]. Several inhibitors of PI3K and IGF1R are currently in Phase I or II clinical trial for the treatment of various solid tumors. Rapamycin, an inhibitor of mTORC1, enhances the antitumor effect of cisplatin in alpha fetoprotein induced gastric cancer, while NVP-BEZ235, which is a dual inhibitor of mTORC1 and PI3K, inhibits T-cell ALL cell lines when combined with other drugs such as cyclophosphamide, cytarabine or dexamethasone [65,92].

Targeting glucose metabolism, which is the central energetic resource of the cell is considered one of the most important anticancer strategies. Selected components of the glycolytic pathway were targeted such as enzymes involved in glucose transport as well as enzymes involved in glucose breakdown. Inhibitors of glucose transporters such as WZB117, Phloretin and Ritonavir demonstrate their anticancer effect through decreased uptake of glucose and thereby lowering the rates of glycolysis [65]. These agents alone or in combination with chemotherapy demonstrated *in vitro* activity against various cancers such as colon cancer, leukemia, lung cancer, breast cancer and multiple myeloma. The non-selective inhibitor of the enzyme hexokinase 2-deoxyglucose has been associated with toxicity in patients with glioma who are treated with radiotherapy at the same time. However, preclinical results obtained with selective agents such as 3-bromopyruvate was promising in rodent tumor models. Pyruvate kinase (PK), which catalyzes the conversion of phosphoenolpyruvate and ADP into pyruvate and ATP has been reported to be associated with tumor growth and cisplatin resistance. Targeting this enzyme with shRNA improved the therapeutic efficacy of cisplatin through increased apoptosis and growth inhibition in *in vitro* studies. Oxamate, a pyruvate analog that inhibits glycolysis by inhibiting the conversion of pyruvate to lactate, has been reported to reverse lactate dehydrogenase associated resistance against paclitaxel (taxol), a widely used chemotherapeutic agent used in the treatment of a variety of human cancers [42,65].

Dichloroacetate (DCA), an inexpensive, orally available drug that is typically used for the treatment of hereditary lactic acidosis, inhibits PDK1, which is often hyper activated in malignant cells. DCA has been reported to exert antineoplastic activity through reactivation of a signal transduction cascade that regulates mitochondrial apoptosis and local invasion in cancer cells. DCA is well tolerated by patients with glioblastoma, and has been reported to exhibit successful outcome in non-Hodgkin’s lymphoma, based on preliminary clinical studies. The anticancer effects of the antidiabetic agent metformin, which targets mitochondrial respiration through interference with mitochondrial complex I is currently being assessed in several cohorts of patients with breast, pancreatic and prostate cancer [42,93].

Rapidly proliferating cancer cells depends on anabolic pathways such as lipid, protein and nucleotide biosynthesis pathways to generate biomass. Cancer cells have been suggested to be auxotrophic for several non-essential amino acids, including asparagine. Therefore, a bacterial variant of L-asparaginase was approved by the FDA for the treatment of acute lymphoblastic leukaemia. Although not completely clear, this enzyme was suggested to reduce the availability of circulating asparagine in cancer cells. Promising preliminary results were also obtained with a pegylated variant of arginine deiminase, which converts circulating L-arginine into L-citrulline. Drugs targeting fatty acid biosynthesis pathway include FASN inhibitors such as cerulenin, C75, orlistat with anti-tumor activity, which were tested *in vitro* and in mice models. Cerulenin and C75 are small molecule inhibitors that induce cancer cell apoptosis, while orlistat has both cytostatic and cytotoxic effects on cancer cells [42,94]. Glutaminolysis, which triggers tumor growth was reportedly inhibited by glutaminase (GLS) inhibitor such as Bis-2-[5-phenylacetamido-1,2,4-thiadiazol-2-yl] ethyl sulphide (BPTES) or siRNA. BPTES slowed the growth of glioblastoma cells that have isocitrate dehydrogenase 1 (IDH1) mutation and also decreased aerobic cell proliferation through hypoxic cell death [65].

In order to study drugs that target nucleotide biosynthesis and the pentose phosphate pathway, molecules that inhibit *de novo* DNA synthesis has been tested in several cancer cells. The level of ribose-5-phosphate (R5P), one of the most important precursor molecules of the nucleotide biosynthesis pathway, were reduced by blocking the rate limiting enzymes of the oxidative branch of pentose phosphate pathway using the inhibitor of G6PDH enzyme, dehydroepiandrosterone (DHEA). Also, reserveratrol and oxythiamine (OT) were used to inhibit the activity of G6PDH and transketolase (TK), respectively, in colon cancer to induce apoptosis [95].

## 5. Conclusions

Cancer is a genetic disease, which progresses in a multistage process, involving both genetic and morphologic transformations. The genetic cause of cancer is grouped into direct and indirect causes. The direct cause includes genetic and epigenetic factors. On the other hand, the indirect cause includes the signaling molecules that may or may not be part of the metabolic process [65]. The molecules associated with signal transduction can be either from the external supply of nutrients such as glucose and glutamine or internal products arising from the metabolism of the external nutrients. The latter group consists of organic acids (e.g., lactic acid), amino acids (e.g., glutamine, arginine, glycine, aspartic acid, *etc*.), lipid biosynthesis products or intermediates (e.g., choline, palmitate, *etc*.), nucleic acid biosynthesis products or intermediates (e.g., ribose-5-phoshpate, *etc*.) and even the reactive oxygen species (ROS) generated from cellular metabolism [55]. While all these molecules are also synthesized by normal proliferating cells, the major difference between cancer cells and proliferating cells is that normal cells have checkpoints, which are regulated by certain regulatory molecules, whereas cancer cells show abnormal/dysregulated metabolism enabling abnormal growth and proliferation of cells.

A key goal in cancer research is to identify altered metabolic pathways or checkpoints that could be targeted to slow or halt growth or initiate death of cancer cells, sparing the normal cells. Conventional chemotherapy drugs kill cancer cells through cytotoxicity, which is also known as apoptosis. There are hundreds of different types of chemotherapy drugs to treat different types of cancer. They are classified based on their chemical nature and mode of action. Most chemotherapeutic drugs interfere with cell cycle progression or induce apoptosis. As a result, the actions of these drugs are non-specific and they can also kill the normal cells surrounding the tumor microenvironment. Due to the non-specific nature of chemotherapy drugs, targeted therapies are preferred over conventional chemotherapy. Targeted therapy is also considered highly important to address the growing problem of chemotherapy resistance in various types of cancer. However, the number of new drugs is still very few, and requires approval from US Food and Drug Administration (FDA). As a result, treatment of cancer is still largely dependent on chemotherapy drug and/or combination therapy. Also, new drug design and development is a costly procedure, and the uses of these drugs require careful assessment of the cancer stage and patients’ health and immune status.

Cancer metabolism is now an area of intensive research for cancer biologists because of its implication in combating emerging drug resistance and novel drug discovery. Because of the complex nature of the disease cancer, the interconnections between signaling pathways and metabolic pathways, and the cascading events that transform a normal cell to a tumor cell, a system-wide approach is often considered highly important in determining the root causes of cancer or in determining the most effective targets for therapy. Systems biology approach is built on combining empirical, mathematical and computational techniques to understand the complex biological and physiological phenomena within cells. Inside the cell, hundreds of proteins are involved in transferring signaling processes to ensure proper functioning of a cell. It is expected that systems biology approach will benefit cancer biology in several ways: (i) identification of prognostic and drug-response biomarkers of tumors; (ii) understanding of molecular mechanisms by building networks and computational models of different stages of cancer progression; and (iii) understanding of the signaling and metabolic networks of metastatic *versus* primary tumors, and improve treatment of the later stages of tumors based on the results of comparative analysis [20]. In other words, systems biology can be used to define signaling network, analyze different steps of oncogenic transformation that is associated with cell behavior and design therapeutic strategies. These approaches are also useful for our understanding of the mechanisms of drug resistance in cancer.

With a variety of metabolic tracers being available, MS-based methods provide sensitive detection of metabolite levels and isotopic enrichment in cells, tissues and biofluids, with the latter representing an informative analyte of minimal invasiveness to humans. Again, in cancer research, there is an intricate relationship between metabolic activity and signaling pathways. Therefore identification of these metabolites is crucial to discover mechanisms of malignancy. Biofluid analysis is restricted to secreted metabolites only, thus limits determination of specific fluxes in central carbon metabolism. To overcome this problem, use of *in vitro* cell culture is highly desirable as it offers high level of control over experimental variables, such as combinatorial use of tracers, nutrient availability, oxygen supply, growth signals, gene expression and stress. Investigation of fluxes that link oncogenic events to aberrant growth and the use of stable isotopes in monocultured cancer cells is therefore a powerful method to elucidate the details of cellular metabolism because fluxes can be evaluated under a wide variety of conditions and thus identify the metabolic targets. However, responses in cell culture cannot be directly extrapolated to *in vivo* conditions. Metabolic fluxes could be more realistically assessed in a tumor xenograft model, in a mammalian host. Nevertheless, isotopic studies with cancer cell lines can at least build the initial framework and pave the way to elucidate the fundamental biochemistry of proliferation *in vivo*.

The by-products or intermediates formed in metabolic pathways could potentially be used as biomarkers for the diagnosis and prognosis of cancer or to assess how cancer cells respond to certain drugs, and thereby evaluate the efficacy of drugs. MFA is currently being applied to investigate how energetic and biosynthetic pathways rewire themselves as a result of oncogenic transformation. However, one of the biggest challenges in unravelling precise metabolic signatures of tumor cells is that heterogeneities exist within the metabolic pathways of different cancer types, or cancers of different tissues, or even cancer cells with thin the same tumor. In general though, the rewiring of metabolic pathways is essentially directed to enhance macromolecular synthesis and ATP production to support rapid proliferation of tumor cells. The understanding of these reconfigured, tumor-associated metabolic pathways has been suggested to be important to identify attractive targets for therapeutic interventions, apart from their diagnostic and prognostic values. It is however, critical to consider possible detrimental effects of inference with these pathways on normally proliferating cells. A number of small molecule drugs that might specifically inhibit key metabolic steps associated with tumor growth are currently at various stages of development. These include drugs that inhibit enzymes that are members of glucose uptake and glycolytic pathways, amino acids biosynthesis, fatty acid biosynthesis, nucleotide biosynthesis as well as signaling pathways that modulate cancer metabolism. Very few of these drugs are now FDA approved for clinical use, but several are at phase I or II clinical trial. While clinical efficacy of these drugs was variable, and some appeared to have unacceptable side effects, several others demonstrated appreciable performance in reversing chemoresistance in cancer. With rapid technological advancement in analytical and computational tools to gather system-wide omics data, it is likely that our understanding of tumor associated metabolic alterations will become more pronounced in the near future facilitating the development of more effective and safer, metabolically targeted, cancer drugs.
